# Cellular Fragments as Biomaterial for Rapid In Vitro Bone-Like Tissue Synthesis

**DOI:** 10.3390/ijms21155327

**Published:** 2020-07-27

**Authors:** Mst Nahid Akhter, Emilio Satoshi Hara, Koichi Kadoya, Masahiro Okada, Takuya Matsumoto

**Affiliations:** Department of Biomaterials, Okayama University Graduate School of Medicine, Dentistry and Pharmaceutical Sciences, 2-5-1 Shikata-cho, Kita-Ku, Okayama-shi, Okayama-ken 700-8558, Japan; pkhe9hyx@s.okayama-u.ac.jp (M.N.A.); gmd421209@s.okayama-u.ac.jp (E.S.H.); de422015@s.okayama-u.ac.jp (K.K.); m_okada@cc.okayama-u.ac.jp (M.O.)

**Keywords:** bone tissue engineering, cell nanofragments, dead cells, mineralization, osteogenesis

## Abstract

Current stem cell-based techniques for bone-like tissue synthesis require at least two to three weeks. Therefore, novel techniques to promote rapid 3D bone-like tissue synthesis in vitro are still required. In this study, we explored the concept of using cell nanofragments as a substrate material to promote rapid bone formation in vitro. The methods for cell nanofragment fabrication were ultrasonication (30 s and 3 min), non-ionic detergent (triton 0.1% and 1%), or freeze-dried powder. The results showed that ultrasonication for 3 min allowed the fabrication of homogeneous nanofragments of less than 150 nm in length, which mineralized surprisingly in just one day, faster than the fragments obtained from all other methods. Further optimization of culture conditions indicated that a concentration of 10 mM or 100 mM of β-glycerophosphate enhanced, whereas fetal bovine serum (FBS) inhibited in a concentration-dependent manner, the mineralization of the cell nanofragments. Finally, a 3D collagen-cell nanofragment-mineral complex mimicking a bone-like structure was generated in just two days by combining the cell nanofragments in collagen gel. In conclusion, sonication for three min could be applied as a novel method to fabricate cell nanofragments of less than 150 nm in length, which can be used as a material for in vitro bone tissue engineering.

## 1. Introduction

Bone is a complex tissue composed of distinct types of highly dynamic cells (e.g., osteoblasts, osteoclasts, osteocytes) and a rich extracellular matrix (ECM) where interactions between organic (e.g., collagen, proteoglycans) and inorganic (e.g., apatite) materials occur, providing the physico-mechanical properties of the skeletal framework in response to the constantly changing shape and composition of the body [1,2,3,4]. The complexity of bone tissue has inspired material scientists, chemists, and biologists to try to develop new methods for bone regeneration or bone tissue synthesis in vitro [1,5,6].

The term organogenesis refers to a developmental process in which tissues and organs are developed from embryonic stem cells [7,8]. The concept of organogenesis for application in tissue engineering has gained great attention recently, because the establishment of novel methods for in vitro organogenesis (or tissue synthesis) may allow unprecedented progress in organ transplantation therapies or drug screening. However, current stem cell-based methods for bone tissue synthesis in vitro usually requires more than 14 to 21 days [9,10,11,12]. The main reason for such long term culture is that stem cells (e.g., mesenchymal stem cells (MSCs)) need to differentiate into mature osteoblasts or chondrocytes, which are the cells promoting mineral formation. A widely accepted concept is that osteoblasts and chondrocytes, when induced to differentiate into mature cells, start to secrete matrix vesicles (MVs), which are known to be the nucleation site for mineral formation [13,14]. In an attempt to promote more rapid bone tissue synthesis, researchers have therefore, customized a combination of stem cells, scaffolds and growth factors (e.g., bone morphogenetic proteins (BMPs)) or alternative biofunctional molecules (e.g., statins, menaquinone-4) [15,16,17,18,19], though all these processes are still time-consuming and costly. Other techniques involve the utilization of pre-osteoblastic or pre-osteocytic cell lines, such as MLO-A5 cells, which can promote bone-like mineral formation within seven days of culture [20]. 

Cell-free approaches for rapid bone-like mineral formation have also been attempted, such as those based on the isolation of osteoblast-secreted MVs [21,22]. Nevertheless, the application of the isolated MVs in bone tissue engineering still faced hindrances, as previous studies were unable to induce mineral formation in vitro using MVs [21,22], or it still required long term culture for mineralization [20]. 

On the other hand, aside from the concept of MV-based mineralization, a previous study demonstrated that dead cells could be a nucleation site for mineral formation [23]. Nevertheless, the mechanisms of how dead cells could be the nucleation site for mineral formation was not clarified. Moreover, a deeper analysis of the process of mineral formation, including its quantitative and qualitative analysis from a Material Science perspective, has not been performed. Therefore, the objective of this study was to explore the concept of using dead cells as materials for bone-like tissue formation in vitro by developing and optimizing the methods to disrupt live cells and fabricate cell nanofragments, and utilize them to induce rapid bone-like tissue synthesis in vitro. The results surprisingly showed that mechanical fragmentation by ultrasonication for three min allowed the fabrication of small and homogeneous nanofragments, which induced markedly rapid mineralization in vitro in just one day.

## 2. Results

### 2.1. Comparative Analysis of the Methods to Obtain Cell Nanofragments

**Figure 1:**
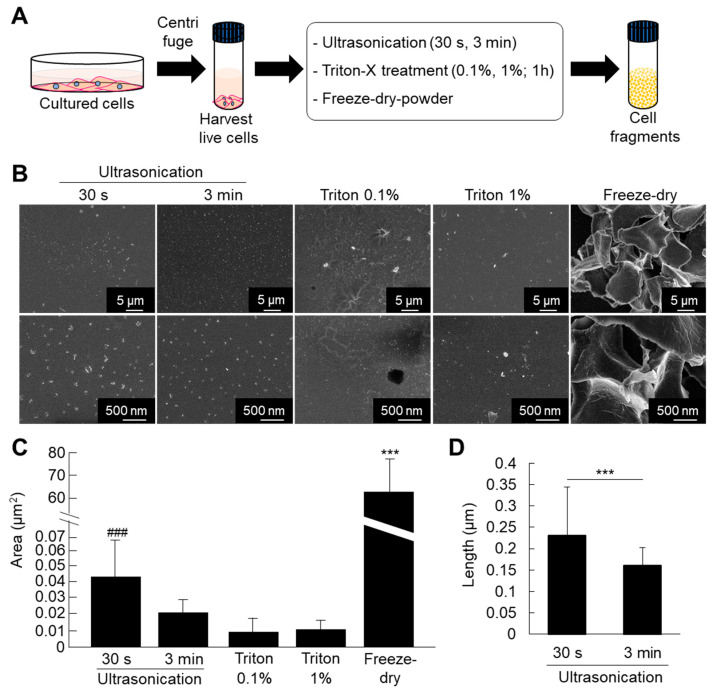
(**A**) Protocol for fabrication of cell fragments by mechanical and chemical methods. (**B**) Ultrastructural analysis of the cell nanofragments obtained by mechanical and chemical disruption of ATDC5 cells, observed by SEM. Note that the cell nanofragments obtained by ultrasonication for 3 min were smaller and more homogenous compared to those obtained by ultrasonication 30 s. Triton-treated (0.1% and 1%) samples showed a thin film-like structure. Freeze-dried powder fragments were large, at the micrometer level. (**C**) Graph shows the quantitative analysis of the average area (µm^2^) of the fragments. Note the high standard deviation in the cell nanofragments obtained by ultrasonication 30 s and freeze-dried powder, representing the heterogeneity of the cell fragment size. *** *p* ≤ 0.001, ANOVA, Bonferroni post-hoc test. Freeze-dried powder samples were significantly larger than all other samples. The size of the cell nanofragments obtained by ultrasonication 30 s was significantly larger compared to ultrasonication 3 min or triton treatments. ### *p* ≤ 0.001, ANOVA, Bonferroni post-hoc test, excluding the freeze-dried powder group in the analysis. (**D**) Graph shows the quantitative analysis of the length (nm) of the cell nanofragments obtained by ultrasonication 30 s or 3 min. The cell nanofragments obtained by ultrasonication 3 min were significantly smaller than those obtained by ultrasonication 30 s. The high standard deviation of the nanofragments obtained by ultrasonication 30 s demonstrates the heterogeneity of the sample.

ATDC5 pre-chondrogenic cells were used for comparative analysis of different methods for fabrication of cell fragments. The cells were submitted to fragmentation by ultrasonication (30 s and 3 min), triton treatment (0.1% and 1%), or freeze-dried powder methods. The results of SEM observation and quantitative analysis showed that the cell nanofragments were smaller and homogenous in the case of ultrasonication for 3 min compared to those obtained by ultrasonication 30 s (Figure 1B–D). Note that the cell nanofragments obtained by 3 min-ultrasonication were of approximately 150 nm in length, while those obtained by ultrasonication 30 s were of 230 nm on average, but with a wide range in size as demonstrated by the high standard deviation (Figure 1C). Triton treatment (0.1% and 1%) allowed the formation of homogeneous cell nanofragments; however, there was a concomitant formation of a film-like structure. On the other hand, the fragments obtained by freeze-dried powder method were heterogeneous in size and at a micrometer scale (Figure 1C). Fine milling using grinding bowls and pestle was also tried as an alternative method, but due to the high lipid contents of the cell nanofragments, they could not be completely harvested as they remained aggregated and adhered to the bowl.

**Figure 2:**
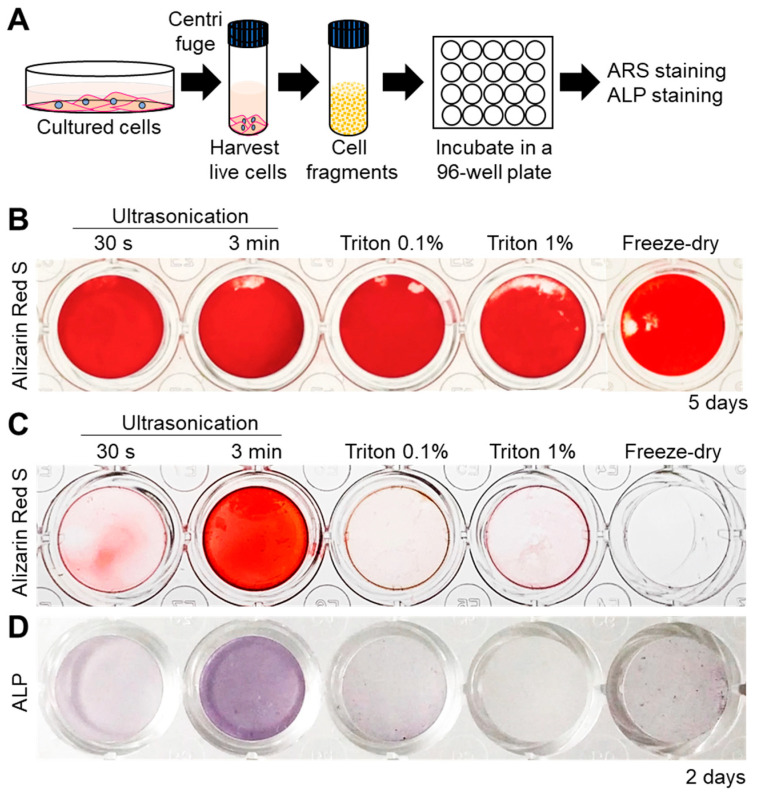
(**A**) Experimental protocol for cell nanofragment mineralization and staining. Cell fragments were obtained from ATDC5 cells by the 5 different methods and incubated at a concentration of 300 μg/mL in α-MEM supplemented with 10 mM β-glycerophosphate (β-GP) for up to 5 days. (**B**) Alizarin Red S (ARS) staining showing that all cell fragments prepared by the 5 different methods mineralized within 5 days. (**C**) Cell nanofragments obtained by ultrasonication 3 min showed markedly faster mineralization (after 2 days) compared to those obtained by the other methods. (**D**) Alkaline phosphatase (ALP) staining showed a high ALP activity in the cell nanofragments obtained by 3 min ultrasonication.

Next, the mineralization ability of all the cell fragments was assessed by incubating them in normal culture medium (α-MEM) supplemented with 10 mM β-Glycerophosphate (β-GP, Figure 2A). Alizarin Red S (ARS) staining for detection of calcium revealed that all cell fragments were able to promote mineral formation within 5 days (Figure 2B). Interestingly, however, mineralization was faster in the case of 3 min-ultrasonicated nanofragments compared to those obtained by all other methods, after 2 days of culture (Figure 2C). The faster mineralization of 3 min-ultrasonicated nanofragments corresponded with a high activity of alkaline phosphatases (ALPs) in the samples (Figure 2D), suggesting that ALPs are crucial enzymes determining the mineralization of the cell nanofragments by cleaving phosphate-containing molecules (e.g., pyrophosphate, phosphoproteins, β-GP) and releasing free phosphate ions that subsequently react with calcium.

**Figure 3:**
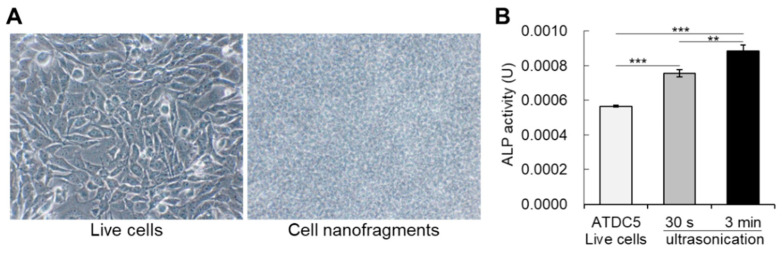
(**A**) Bright-field images of live cells (ATDC5 cells) on the left, and the cell nanofragments on the right side. (**B**) Graph shows the alkaline phosphatase enzymatic activity in live cells, as well as in the cell nanofragments immediately after cell disruption by ultrasonication for 30 s or 3 min. Note that cell disruption by ultrasonication increases the activity of alkaline phosphatases, and is significantly higher in the cell nanofragments obtained from ultrasonication 3 min. ** *p* ≤ 0.01 and *** *p* ≤ 0.001, ANOVA, Bonferroni post-hoc test.

To further evaluate whether cell fragmentation could affect ALP activity, we performed a comparative analysis of ALP enzymatic activity between live cells (Figure 3A) and the immediately-fabricated nanofragments by ultrasonication 30 s and 3 min. As shown in Figure 3B, disruption of cells significantly enhanced the ALP enzymatic activity, in a ultrasonication time-dependent manner. These results support the notion of the existence of a mechanism regulating ALP enzyme activity in intact live cells, which can be manipulated by cell fragmentation.

**Figure 4:**
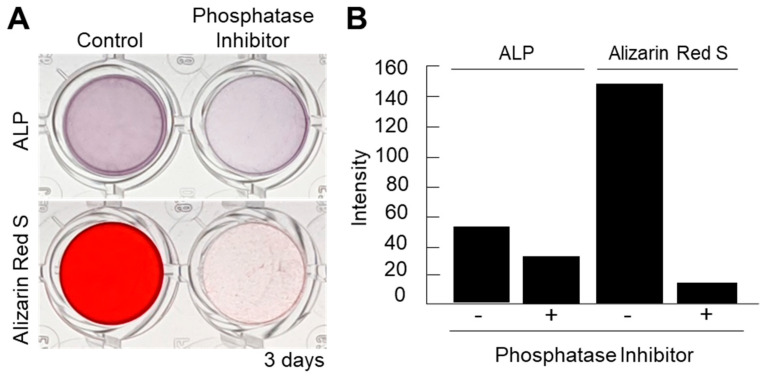
(**A**) Inhibition of ALP activity using a phosphatase inhibitor cocktail partially suppressed the ALP activity but completely blocked the mineralization of the cell nanofragments, as determined by ARS staining. Alpha-MEM was used as the vehicle in the inhibition assay. (**B**) Quantitative analysis of ALP and ARS staining performed with ImageJ. A blank well was used as control for the image analysis.

Next, in order to confirm the importance of ALPs in the mineralization of cell nanofragments, a loss-of-function experiment was performed with a phosphatase inhibitor cocktail. As shown in Figure 4A,B, the phosphatase inhibitors could completely suppress the cell nanofragment-based mineralization, demonstrating that the minerals formed from the cell nanofragments are based on the activity of cellular phosphatases, and not a spontaneous precipitation of minerals.

### 2.2. Optimization of Culture Condition of Nanofragment Mineralization and Characterization of the Minerals

**Figure 5:**
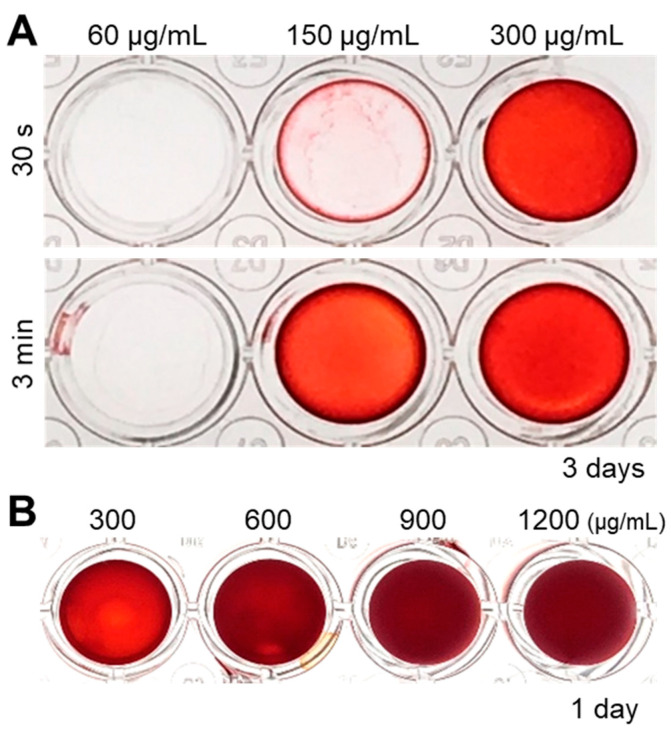
(**A**) Analysis of the optimal concentration of cell nanofragments obtained from ATDC5 cells. Cell nanofragments at different concentrations (60 μg/mL, 150 μg/mL, and 300 μg/mL) were incubated in α-MEM supplemented with 10 mM β-glycerophosphate (β-GP) for 3 days. ARS staining demonstrates the rapid mineralization of the cell nanofragments obtained by 3 min ultrasonication, compared to those obtained by ultrasonication 30 s. After 3 days of incubation, the concentration of 150 μg/mL (ultrasonication 3 min) was the minimum amount of nanofragments necessary to obtain a thin and homogeneous mineralized layer. (**B**) ARS staining of cell nanofragments incubated for 1 day at concentrations above 300 μg/mL. The concentration of 300 μg/mL was the minimum amount of nanofragments required to obtain a thin and homogeneous mineralized layer after 1 day of incubation. Concentrations above 300 μg/mL showed a thick layer, which could be retaining small amounts of the ARS compound inside.

Since ultrasonication allowed the fabrication of homogeneous cell nanofragments, we further evaluated the optimal concentration of the cell nanofragments to induce rapid in vitro mineralization. As shown in Figure 5A, a concentration of at least 150 μg/mL of cell nanofragments was required to obtain initial mineral deposition with the cell nanofragments obtained by ultrasonication 3 min. On the other hand, mineralization of cell nanofragments fabricated by ultrasonication 30 s could not be clearly detected at a concentration of 150 μg/mL, but was prominent at a concentration of 300 μg/mL, after 3 days of incubation. Higher concentrations of the cell nanofragments could induce even more rapid mineralization within 24 h, although there was a formation of a thick mineralized film due to the high amount of the nanofragments (Figure 5B). Therefore, the minimum amount of cell nanofragments that was able to induce mineralization in a thin and homogeneous layer was 300 μg/mL.

**Figure 6:**
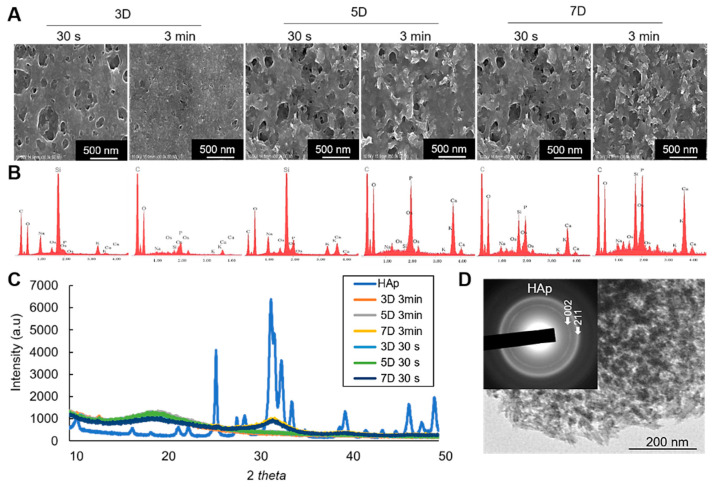
(**A**) SEM images of the mineralized cell nanofragments without NaClO treatment. (**B**) EDX analysis showing the incubation period-dependent increase in calcium and phosphate levels. Note that the amount of calcium in the nanofragments obtained by ultrasonication 3 min was higher compared to ultrasonication 30 s, at all time points; 3D: 3 days, 5D: 5 days, 7D: 7 days. (**C**) XRD analysis of the precipitated products (minerals), which were identified to be amorphous calcium phosphate (3D and 5D samples) or hydroxyapatite (7D samples) in the cell nanofragments obtained by either 30 s or 3 min. Commercially available hydroxyapatite (HAp) was used as a reference control. (**D**) TEM image and electron diffraction analysis of the minerals formed from the cell nanofragments after 7 days of incubation. The cell nanofragments were collected from ATDC5 cells and incubated in α-MEM supplemented with 10 mM β-GP for up to 7 days.

For characterization of the minerals, scanning electron microscopic (SEM) observation shows the morphological characteristics of the mineralized cell nanofragments, and energy dispersive X-ray spectroscopic (EDX) analysis confirmed the presence of calcium in the cell nanofragments incubated for more than 3 days (Figure 6A,B). Note, however, that the calcium levels detected in the cell nanofragments obtained by ultrasonication 30 s after 3 days of incubation was low (Figure 6B), which is consistent with the difference in the mineralization ability between the cell nanofragments from 30 s and 3 min shown in Figure 2C and Figure 5A. Note also the increase in calcium peak levels in the cell nanofragments as the incubation period increases to 5 and 7 days.

On the other hand, the results of X-ray diffraction (XRD) analysis identified the initial minerals to be amorphous calcium phosphate (3 and 5 days), which then crystallized into hydroxyapatite (HAp) after 7 days of incubation (Figure 6C). Note the broad peak in the 31–33° 2θ region characteristic of the 211, 112, and 300 planes of HAp, suggesting the low crystallinity of the HAp product formed from the cell nanofragments incubated for 7 days. The same pattern of crystalline phase change was observed in the cell nanofragments obtained by ultrasonication 30 s and 3 min (Figure 6C). Further qualitative analysis of the minerals by transmission electron microscopy (TEM)/electron diffraction confirmed that they were HAp (Figure 6D, insert).

**Figure 7:**
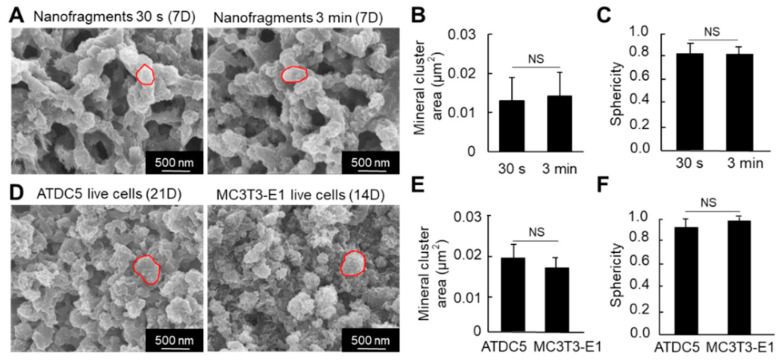
(**A**) SEM images of the mineral structures formed from cell nanofragment mineralization after treatment with NaClO to remove all organic material. (**B**,**C**) Graphs show the mineral cluster area (**B**) and the sphericity (**C**) of the minerals formed by the mineralization of cell nanofragments obtained by either 30 s or 3 min of ultrasonication. (**D**) SEM images of the mineral structures formed from ATDC5 and MC3T3-E1 live cells cultured for 21 and 14 days, respectively, in mineralization-inducing conditions. The minerals were treated with NaClO to remove all organic material. (**E**,**F**) Graphs show the mineral cluster area (**E**) and the sphericity (**F**) of the minerals formed by the mineralization of ATDC5 and MC3T3-E1 live cells. Note that the size and quasi-spherical shape of the minerals formed from the cell nanofragments were comparable to those formed by ATDC5 and MC3T3-E1 live cells. NS = non-significant, Student’s *t*-test.

Regarding the size of the HAp minerals (calcospherites), there was no significant difference in the size (area) between the nanofragments obtained by 30 s and 3 min sonication, after 7 days of incubation (Figure 7A,B). Note that most of the minerals were quasi-spherical, with sphericity values above 0.82 (Figure 7C). 

We then compared the nanostructure of the minerals formed from the cell nanofragments with those formed from ATDC5 and MC3T3-E1 osteoblastic cells cultured in mineralization-inducing conditions. As shown in Figure 7D, the minerals formed from the cell nanofragments showed no significant difference in the size and shape (sphericity) of those formed from the ATDC5 and MC3T3-E1 live cells.

**Figure 8:**
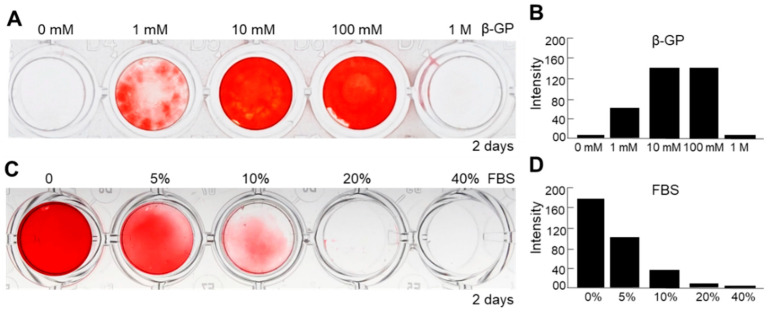
Analysis of factors affecting the mineralization of ATDC5-derived cell nanofragments (300 μg/mL). (**A**,**C**) ARS staining of cell nanofragments incubated for 2 days with increasing doses of β-glycerophosphate (β-GP, (**A**)) or fetal bovine serum (FBS, (**C**)). (**B**,**D**) Graphs show the quantitative analysis of ARS staining performed with ImageJ. Note that β-GP at concentrations of 10 mM or 100 mM of enhanced (**A**,**B**), while fetal bovine serum (FBS) inhibited in a dose-dependent manner (**C**,**D**), the mineralization of nanofragments. Beta-GP at a concentration of 1 mM partially promoted the mineralization of nanofragments. Alpha-MEM was used as the vehicle in the experiments. In the studies with different concentrations of FBS, 10 mM β-GP was added in all samples.

To further optimize the methods of culture condition of nanofragment mineralization, we evaluated the effect of β-GP, which is known to be a substrate of mineralization-associated phosphatases for the release of free phosphate ions [24], as well as that of fetal bovine serum (FBS), which is essential for cell culture but has been reported to have controversial effects on osteogenesis [25,26,27], on the mineralization of cell nanofragments obtained by sonication 3 min. Cell nanofragments obtained by 3 min ultrasonication were incubated in gradient concentrations of β-GP and FBS. As shown in Figure 8, concentrations of 10 mM or 100 mM markedly enhanced (Figure 8A,B), whereas FBS inhibited in a concentration-dependent manner (Figure 8C,D) the mineralization of the cell nanofragments. The results indicated that the optimal condition for nanofragment mineralization was with 10 mM β-GP supplementation, and without FBS.

**Figure 9:**
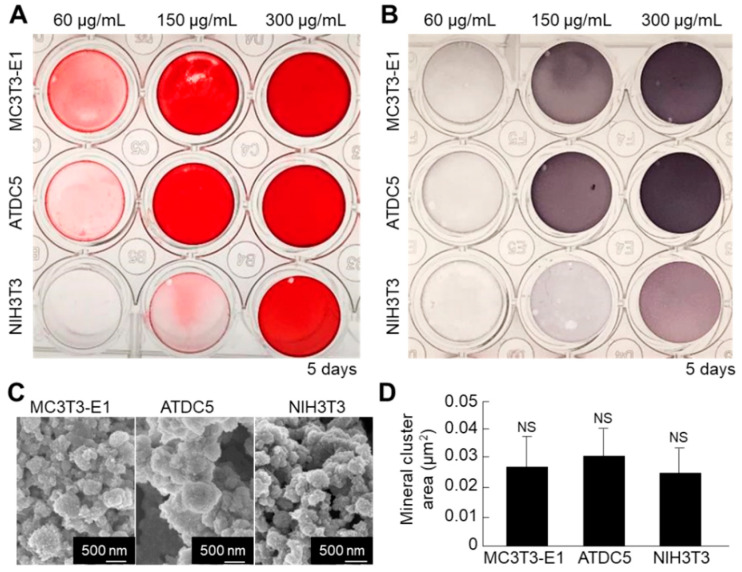
(**A**,**B**) Comparative analysis of mineralization ability of cell nanofragments from three different types of cells (ATDC5 pre-chondrogenic cells, MC3T3-E1 osteoblastic cells, and NIH3T3 fibroblasts). ARS (**A**) and ALP staining (**B**) of the nanofragments incubated in α-MEM supplemented with 10 mM β-glycerophosphate for 5 days. Note a more rapid mineralization ability of the cell nanofragments derived from MC3T3-E1 and ATDC5 cells, compared to those from NIH3T3 fibroblasts. Note also that even non-mineralizing cells (fibroblasts) are able to promote mineralization after being fragmented. (**C**) SEM images of the mineral structures formed from nanofragments from the three cell types, after treatment with NaClO. (**D**) Graph shows the quantitative analysis of the mineral cluster area, indicating a similar size of minerals independently of the cell source. NS = non-significant, one-way ANOVA.

Next, we compared the mineralization ability between the cell nanofragments obtained from ATDC5 cells and those from MC3T3-E1 osteoblastic cells and NIH3T3 fibroblasts, in order to determine the optimal cell source. As shown in Figure 9A,B, the cell nanofragments from MC3T3-E1 and ATDC5 cells mineralized more rapidly than those from NIH3T3 fibroblasts, as demonstrated by ARS staining, which corresponded with a higher ALP activity in the samples. Quantitative analysis of the mineral size showed no statistically significant difference among the cell nanofragments from the three cell types, despite that the minerals formed by ATDC5 cell nanofragments were apparently larger (Figure 9C,D). 

### 2.3. Utilization of Cell Nanofragments for 3D Bone-Like Tissue Synthesis In Vitro

**Figure 10:**
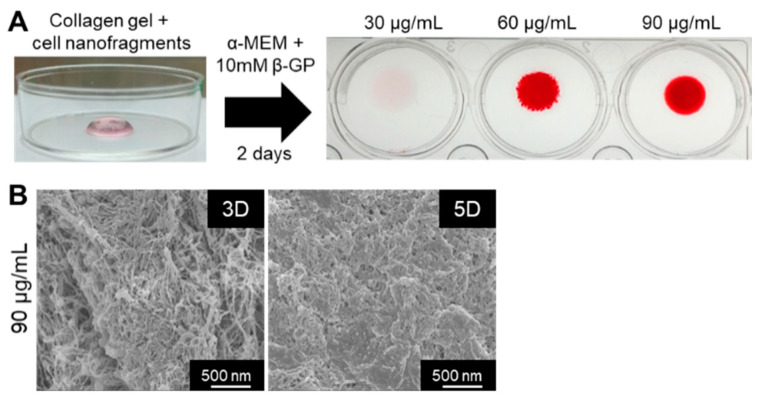
(**A**) Fabrication of a 3D collagen-cell nanofragment-mineral complex mimicking a bone-like structure using ATDC5 cell-derived nanofragments and collagen gel. The collagen-cell nanofragment-complex can mineralize in just 2 days with cell nanofragment concentrations of 60 µg/mL and 90 µg/mL, but not at a concentration of 30 µg/mL. (**B**) SEM images of the 3D collagen-cell nanofragment-mineral complex mimicking a bone-like structure. Note the deposition of minerals onto the collagen fibers after 3 days (3D), and the densely compacted minerals after 5 days of incubation in α-MEM supplemented with 10 mM β-GP.

Finally, we tested whether the cell nanofragments could be used as a biomaterial for 3D bone-like tissue synthesis. As shown in Figure 10A, ATDC5-derived cell nanofragments were mixed with collagen gel for the fabrication of a 3D collagen-cell nanofragment-mineral complex structure, which was achieved in just 2 days with cell nanofragment concentrations of 60 µg/mL and 90 µg/mL, but not at a concentration of 30 µg/mL. Further analysis of the collagen-cell nanofragment-mineral complex microstructure by SEM showed in detail the mineral deposition onto the collagen fibers after 3 days, and a high mineralized structure after 5 days of incubation (Figure 10B). These results indicate that the cell nanofragments can be novel tools for fabrication of 3D structures of diverse shapes which can mimic a bone-like structure.

## 3. Discussion

Bone tissue plays critical roles in the body, such as locomotion, ion supply, and protection of organs, including the brain, heart, and the marrow. Genetic diseases associated with defective bone formation (e.g., fibrodysplasia ossificans progressiva, hypophosphatasia) or with incurable bone resorption (e.g., osteoporosis) cause significant impairment in the individual’s quality of life and are increasingly demanding for new therapies. Moreover, the bone tissue is also the target of multiple types of cancer metastasis. In this context, in vitro bone synthesis can be considered as one of the fundamental targets in the tissue engineering field, because the development of devices including more sophisticated bone-on-chips, can allow, for instance, a facile and systematic screening of novel drugs for such diseases.

Nevertheless, despite its importance, the synthesis of bone tissue in vitro has been still a great challenge. The development of methods to fabricate bone tissue still faces significant hindrances mainly due to the remaining controversies regarding the understanding of the exact process of in vivo bone formation. For instance, important reports have shown the determinant roles of lipids and matrix vesicles in initial bone formation [26,27]. However, current methods to fabricate bone tissue generally involve live cells and require approximately two to three weeks. Therefore, in vitro osteogenic conditions are unable to mimic the in vivo conditions, not only regarding the composition but also in terms of the micro-environmental conditions (e.g., pH, osmotic pressure, mechanical pressure) [28]. 

In this study, based on the fact that dead cells were shown to be nucleation site for mineral precipitation [29], we pulverized live cells to fabricate cell fragments to induce rapid mineralization in vitro, which could be achieved in one to two days. 

The mechanisms of dead-cell or cell nanofragment-induced mineral formation are still unclear. Previous reports have demonstrated that genomic DNA can mineralize [30,31]. These reports present the hypothesis that the phosphate groups composing the DNA backbone can be cleaved by phosphatases for the release of free phosphate ions that subsequently react with calcium, and form calcium phosphate minerals [32]. On the other hand, our previous study demonstrated that the plasma membrane nanofragments can be the nucleation site for mineral formation in vivo, during the initial steps of endochondral ossification, as well as in vitro, by isolating only the plasma membrane fraction from cultured cells [33,34]. Therefore, when using the cell nanofragments, genomic DNA and plasma membrane would possibly be the key elements promoting mineralization. Additionally, phosphoproteins in the plasma can also be strong candidates participating in the mineralization process of the cell nanofragments.

Another interesting finding was the fact that cell fragmentation was not the only major determinant of nanofragment mineralization. Ultrasonication for 30 s and 3 min could induce cell fragmentation and release of intracellular mineralizing factors, but the mineralization was more rapid with 3 min ultrasonicated nanofragments, which showed a significantly smaller size (150 nm in length). In other words, the size of the nanofragments was one of the crucial factors determining the nanofragment mineralization, which was related to a prominently higher alkaline phosphatase activity, either immediately after live cell disruption or after incubation in culture medium. Although the exact mechanisms that could trigger the activation of ALPs are unclear, it could be hypothesized that the cell nanofragments smaller than 150 nm in size could present possible conformational changes in their three-dimensional structure, which consequently could show improved affinity or reactivity with the substrates.

The combination of cell nanofragments with materials (e.g., hydrogels, polymers) may also boost the development of novel devices for synthesis of bone tissue in vitro, or fabrication of more sophisticated bone tissue-on-chips [35], which are still on high demand for more efficient drug screening systems or analysis of bone tissue dynamics during physiological or pathological (e.g., metastasis) conditions [36,37,38,39]. In this study, cell nanofragments were combined with collagen gel to fabricate a 3D collagen-cell nanofragment-mineral complex mimicking the bone structure. Further optimization of these scaffolds, with a possible combination of cells, may further allow the development of a 3D bone-like tissue consisting of osteoblasts, osteocytes and even osteoclasts, which can allow a more detailed analysis of the function and behavior of these cells in an environment that can mimic the in vivo conditions. 

In summary, the results of this study demonstrated that ultrasonication for three min was the optimal method for fabrication of cell nanofragments of less than 150 nm in size, which mineralized in just one day in culture medium supplemented with 10 mM β-GP, but without FBS. Therefore, cell nanofragments of less than 150 nm could be used as a biomaterial for rapid synthesis of bone-like tissue in vitro.

## 4. Materials and Methods

### 4.1. Cell Culture and Fabrication of Cell Nanofragments

ATDC5 pre-chondrogenic cells were maintained in Dulbecco’s Modified Eagle Medium (D-MEM) /F12 (Wako Pure Chemical Industries, Osaka, Japan) containing 10% fetal bovine serum (FBS; Life Technologies, Gaithersburg, MD, USA) and 1% penicillin and streptomycin (Sigma-Aldrich, St. Louis, MO, USA), at 37 °C, 5% CO_2_ and 99% humidity. MC3T3-E1 osteoblastic cells were cultured under the same conditions, except for the culture medium, which was alpha Modified Eagle Medium (α-MEM, Wako Pure Chemical Industries). NIH-3T3 fibroblasts were cultured in similar conditions of ATDC5 cells, except for the serum, which was fetal calf serum (Life Technologies). 

For fabrication of cell nanofragments, the cells were first expanded in 225 cm^2^ flasks until confluency. The cells were then trypsinized, collected by centrifugation at 4× *g*, resuspended in culture medium and aliquoted in amounts of 1 × 10^7^ cells in 1.5 mL tubes. The aliquoted cells were then centrifuged at 4× *g* to harvest the cells, and the culture medium (supernatant) was aspirated. The cells were then: 1—resuspended in 1 mL of Milli-Q ultrapure water and submitted to ultrasonication (VP-5S, Taitec, Saitama, Japan) for 30 s or 3 min (with cooling intervals of 30 s after every 30 s of ultrasonication) on ice; 2—resuspended in 1 mL of triton 0.1% and 1% *v*/*v* diluted in Milli-Q ultrapure water and maintained for 1 h; or 3—frozen at −80 °C and vacuum dried. After that, the freeze-dried cells were minutely powdered/pulverized with a surgical blade (#11, Pfizer, New York, NY, USA) and resuspended in 1 mL of Milli-Q ultrapure water. 

The dry weight of 1 × 10^7^ cells was approximately 3 mg, and therefore, the concentration (*m*/*v*) of the cell nanofragments immediately after resuspension was 3 mg/mL, which was further utilized in the experiments at different dilutions.

For the experiments with concentrations of cell nanofragments above 600 mg/mL, the number of cells was increased proportionally. For the experiments with different cell types, due to the differences in the cell size, the dry weight of 1 × 10^7^ cells was measured before each experiment, and the amount of Milli-Q ultrapure water for cell nanofragment suspension was normalized to obtain an equal concentration of 3 mg/mL.

### 4.2. Mineralization Assay

For mineralization assay, cell nanofragments were incubated with α-MEM supplemented with 10 mM β-glycerophosphate (β-GP, Sigma-Aldrich), unless otherwise mentioned, in 48-well or 96-well tissue culture plates for different time intervals (1 to 7 days), at 37 °C, 5% CO_2_, and 99% humidity. After incubation, the samples were then fixed with 4% paraformaldehyde (PFA) without PBS overnight, and gently washed with distilled water (DW) before staining. Mineralization was confirmed by 1% Alizarin Red S (ARS) staining (Sigma-Aldrich), as reported [40]. Briefly, after fixation and washing, the samples were incubated with 1% ARS solution for 10 min, and thoroughly washed (at least 3 times) to remove stain excess. 

Alkaline phosphatase (ALP) staining was performed by incubating the cell nanofragments with 5-bromo-4-chloro-3-indolyl-phosphate and nitro blue tetrazolium (BCIP/NBT) substrate solution (Roche, Basel, Switzerland) for at least 20 min. The samples were then washed gently 3 times to remove the staining substrate. Photographs were taken, and the intensity of the staining was calculated using ImageJ.

### 4.3. Alkaline Phosphatase (ALP) Enzyme Activity Assay

To compare the ALP enzymatic activity between live cells and ultrasonicated cell nanofragments, ATDC5 cells were cultured until confluency, trypsinized, harvested by centrifugation, and resuspended in basal culture medium. A total of 2 × 10^6^ cells were aliquoted in a 1.5 mL tube and centrifuged. The basal medium (supernatant) was then aspirated and replaced with 500 μL of Milli-Q ultrapure water. The cells were then submitted to ultrasonication for 30 s or 3 min on ice, and 500 μL of a p-nitrophenyl phosphate (pNPP) solution containing 1.0 mg/mL pNPP, 0.2 M trizma buffer and 5 mM magnesium chloride (SIGMAFAST™ tablets, Sigma-Aldrich) was added to the tube containing the freshly-prepared cell nanofragments.

In the case of live cells, after centrifugation, they were kept on ice without the addition of ultrapure water in order to avoid cell lysis due to the difference in osmotic pressure. The 1:1 diluted pNPP solution was added to the tube containing the live cells at the same time the pNPP solution was mixed with the freshly-prepared cell nanofragments.

After incubation for approximately 10 min at room temperature, 100 μL of the water-soluble yellowish hydrolyzed product of each sample was transferred into a 96-well plate, and 25 μL of 3 M sodium hydroxide solution was immediately added into each well to stop the reaction. The intensity of the colorimetric substrate was measured by a microplate reader (FlexStation 3 Multi-Mode Microplate Reader, San Jose, CA, USA) at the absorbance of 405 nm wavelength, available at the Central Research Laboratory, Okayama University Medical School. Experiments were performed in duplicate or triplicate samples.

The alkaline phosphatase enzyme activity in the live cells and in the cell nanofragments was estimated based on the concentration of the standard enzyme (5 U/μL, BioDynamics Laboratory Inc., Tokyo, Japan).

### 4.4. Inhibition of Cell Nanofragment Mineralization

For the inhibition assay, ATDC5 cell nanofragments (300 µg/mL) were incubated with 1 μL of phosphatase inhibitor cocktail-2 (Sigma-Aldrich) within 100 μL of α-MEM supplemented with 10 mM β-GP into a 96-well tissue culture plate for 3 days. The phosphatase inhibitor cocktail-2 contains sodium orthovanadate, which inhibits a number of ATPases, protein tyrosine phosphatases, and other phosphate-transferring enzymes; sodium molybdate, which inhibits acid and phosphoprotein phosphatases; sodium tartrate, which inhibits acid phosphatases; and imidazole, which inhibits alkaline phosphatases. After incubation, the samples were fixed with 4% PFA overnight, gently washed with DW, and stained with ARS and ALP staining solutions.

### 4.5. Live Cell Differentiation and Mineralization

For comparative analysis between the minerals formed from live cells and those formed from the cell nanofragments, MC3T3-E1 and ATDC5 cells were induced to differentiate into mature osteoblasts and chondrocytes, respectively. MC3T3-E1 were seeded in 6-well plates at a concentration of 5 × 10^5^ cells and maintained in basal culture medium until confluency. The medium was then replaced with osteogenic medium (basal culture medium supplemented with 100 nm dexamethasone, 10 mM β-GP and 1 μM of ascorbic acid), and the cells were cultured for 2 weeks, with the medium being replaced every second or third day.

ATDC5 cells were seeded at a density of 6000 cells/cm^2^ in a 6-well plate and cultured until confluency. The medium was then replaced with mineralization-inducing medium consisted of basal culture medium supplemented with 5 μg/mL insulin, 5 μg/mL transferrin and 5 ng/mL selenious acid by a 1:1000 dilution of ITS premix Universal Culture Supplement (Corning Inc, Corning, NY, USA), 10 mM β-GP and 50 μg/mL of ascorbic acid. The cells were cultured for 3 weeks, with the medium being changed every second or third day, as reported previously [41].

After incubation, the medium was aspirated, and 1 mL of sodium hypochlorite was added into the wells containing the cells. The solution was transferred into a 1.5 mL tube and kept at room temperature overnight for complete elimination of the organic material. The remaining minerals were then centrifugally-washed 3 times at 12,000 rpm for 5 min with Milli-Q ultrapure water. The obtained minerals were then dispensed onto an aluminum holder and vacuum-dried before SEM observation.

### 4.6. In Vitro Fabrication of a 3D Collagen-Cell Nanofragment-Mineral Complex

For fabrication of a 3D collagen-cell nanofragment-mineral hybrid system mimicking a bone tissue structure, ATDC5 cell nanofragments at concentrations of 30 µg/mL, 60 µg/mL, or 90 µg/mL were mixed within a collagen hydrogel (Cellmatrix type I-A, Nitta gelatin, Osaka, Japan), which was set by mixing with a neutralizing buffer (0.05 N NaOH, 2.2% NaHCO_3_, 200 mM HEPES), according to the manufacturer’s protocol. The gel was then maintained inside the incubator for at least 20 min for complete gelation, and then incubated in α-MEM containing 10 mM β-GP to induce mineralization at 37 °C, 5% CO_2_, and 99% humidity. After 2 days of culture, the collagen-cell nanofragment-mineral complex was collected, fixed with 4% PFA overnight, washed with DW, and stained with 1% ARS solution. The samples were thoroughly washed before being photographed.

The collagen-cell nanofragment-mineral complex was also incubated for 3 days or 5 days for observation of the microstructure by SEM. Briefly, after incubation, the collagen-cell nanofragment-mineral complex was fixed with 2% PFA and 2% glutaraldehyde for at least 1 h, washed with Milli-Q ultrapure water and further fixed with 2% osmium tetroxide for 1 h. The samples were then washed with Milli-Q ultrapure water two times, dehydrated through a grading series of ethanol (70%, 80%, 90%, and 99.8%) and tert-butanol. The samples were then frozen at −80 °C and vacuum-dried.

### 4.7. Scanning Electron Microscopy (SEM) and Energy Dispersive X-Ray Spectroscopy (EDX)

For SEM observation of the size and shape of cell fragments, cultured live cells were disrupted by the 5 different methods described in Section 4.1, and the freshly-obtained fragments were fixed with 2% PFA and 2% glutaraldehyde for at least 1 h, washed with Milli-Q ultrapure water and further fixed with 1% osmium tetroxide (TAAB Laboratories Equipment Ltd., Berkshire, UK) for 1 h before being dispensed onto an aluminum holder.

For observation of the mineralized cell nanofragments and elemental analysis with EDX, the cell nanofragments incubated for 3, 5, or 7 days were collected in 1.5 mL tubes, centrifugally-washed 2 times with Milli-Q ultrapure water and dispensed onto a microscope cover glass fixed on an aluminum holder, and vacuum-dried.

For observation of the size and shape of calcium phosphate minerals, in vitro mineralized cell nanofragments were treated with sodium hypochlorite (Wako Pure Chemical Industries) for at least 5 min to remove all organic material, centrifugally-washed with Milli-Q ultrapure water at 12.000 rpm using a benchtop centrifuge and dispensed onto a microscope cover glass fixed on an aluminum holder, and vacuum dried.

For observation of mineral structure inside the collagen gel, the freeze-dried collagen-cell nanofragment-mineral complexes were torn in half using fine tweezers and fixed onto the aluminum holder with carbon conductive double-faced adhesive tapes (TAAB Laboratories Equipment Ltd.).

After the samples were fixed onto the aluminum holders, they were coated using an osmium coater at an electrical discharge current of 10 mA and a degree of vacuum of 10 Pa for 10 s, and observed using a scanning electron microscope (JSM-6701F, JEOL, Tokyo, Japan). EDX analysis was performed with an SEM (S-4800, Hitachi, Tokyo, Japan) equipped with an X-ray silicon drift detector (EDAX Genesis APEX2, AMETEK Co., Ltd., Berwyn, PA, USA) available at the Central Research Laboratory, Okayama University Medical School.

### 4.8. X-Ray Diffraction (XRD)

For qualitative analysis of the minerals formed from the cell nanofragments, ATDC5 cell nanofragments (300 µg/mL) obtained by 3 min ultrasonication were incubated in 1.5 mL tubes containing α-MEM supplemented with 10 mM β-GP for different time periods (3 to 7 days). Subsequently, the mineralized cell nanofragments were centrifugally-washed with Milli-Q ultrapure water twice at 12,000 rpm using a benchtop centrifuge, dispensed as a thin film onto a non-reflecting silicon plate and vacuum-dried. XRD analysis was performed with RINT2500HF instrument (Rigaku Corp., Tokyo, Japan) at an incidence angle of 1° using Cu-Kα (1.54 Å) irradiation at 40 kV and 200 mA. The XRD measurements were conducted from 10° to 50° at a scan speed of 0.02° min−1. Analysis was performed with triplicate samples. Commercially available hydroxyapatite (HAp) was used as the reference sample.

### 4.9. Transmission Electron Microscopy (TEM) Observation and Electron Diffraction Analysis

For characterization of hydroxyapatite by TEM/electron diffraction, ATDC5 cell nanofragments obtained by 3 min ultrasonication were incubated for 7 days in α-MEM supplemented with 10 mM β-GP. The mineralized cell nanofragments were then centrifugally-washed at 12,000 rpm for 5 min using Milli-Q ultrapure water, at least two times. Mineral precipitations were then spread out onto the TEM grid and vacuum-dried. Samples were observed by a high-resolution TEM (JEM-2100, JEOL, Tokyo, Japan) operated at an accelerating voltage of 200 kV.

### 4.10. Image Analysis

Analyses of nanofragment and mineral sizes and sphericity [42] were performed with ImageJ software (NIH, Bethesda, MD, USA). For quantitative analysis of the minerals, mineral clusters (calcospherites) were delineated and the surface areas and sphericity were then measured. Quantitative analysis was based on the average and standard deviation of at least 20 nanofragments or calcospherites.

### 4.11. Statistical Analysis

Analysis of the differences between groups was performed with unpaired Student’s *t*-test, or one-way ANOVA followed by Bonferroni post-hoc correction test when appropriate. Statview software (version 5.0; SAS Institute Inc., Cary, NC, USA) was used for the analyses. The level of significance was set as *p* ≤ 0.05.

## 5. Conclusions

Together, the results of this study demonstrated that ultrasonication for 3 min was the optimal method for fabrication of cell nanofragments of less than 150 nm in size, which mineralized in just 1 day in culture medium supplemented with 10 mM β-GP, but without FBS. Therefore, cell nanofragments of less than 150 nm could be used as a material for rapid synthesis of bone-like tissue in vitro.
